# Odontogenic brain abscess caused by *Porphyromonas gingivalis* and *Streptococcus constellatus*: a case report and review article

**DOI:** 10.1080/20002297.2025.2485197

**Published:** 2025-04-14

**Authors:** Siyu Sun, Rui He, Shan Chen, Jing Ren, Xinrong Ma, Junying Yang

**Affiliations:** Department of Stomatology, The First Affiliated Hospital, Sun Yat-sen University, Guangzhou, China

**Keywords:** Brain abscess, periodontitis, *Porphyromonas gingivalis*, *Streptococcus constellatus*, mNGS

## Abstract

**Background:**

Odontogenic brain abscess is a rare, but potentially fatal, central nervous system infection, with insidious onset and unclear etiology.

**Methods:**

This case reports a 70-year-old male patient who developed an odontogenic brain abscess secondary to periodontal infection and underwent neurological surgery. Extract pus during surgery for the metagenomic next-generation sequencing (mNGS).

**Results:**

The mNGS of pus samples obtained from brain abscess aspiration identified the periodontal pathogens *Porphyromonas gingivalis* and *Streptococcus constellatus*. Consequently, he was referred to the department of stomatology for further examination and treatment.

**Conclusions:**

Our study found that major periodontal pathogens including *P. gingivalis* and *S. constellatus* were essential in the development of odontogenic brain abscesses; thus, timely intervention and preventive measures are important for treatment.

## Background

A brain abscess is a severe cranio-cerebral infectious disease characterized by focal areas of suppuration within the brain parenchyma [1,2]. It has an annual incidence of 0.90 cases per 100,000 people [3]. The most common etiology is bacterial dissemination from a distant primary site, with the oral cavity being a significant potential source of primary infection [4]. However, the exact mechanism remains unknown [5]. Here, we report a case of odontogenic brain abscess resulting from periodontal infection in a 70-year-old male patient, with the causative organisms identified through macrogenomic second-generation sequencing (mNGS).

## Case report

On 23 December 2023, a 70-year-old Chinese male presented to the First Affiliated Hospital of Sun Yat-sen University with sudden onset of left-sided limb weakness and slurred speech, which had occurred 6 hours prior. He had also been experiencing fever and diffuse headache for the past two days, with a maximum temperature of 38.9°C. The initial physical examination showed a body temperature of 36.6°C, respiratory rate of 18/minute, pulse rate of 80/minute, and blood pressure of 106/72 mmHg. He denied any history of trauma, systemic diseases, or drug allergies.

**Figure 1.**
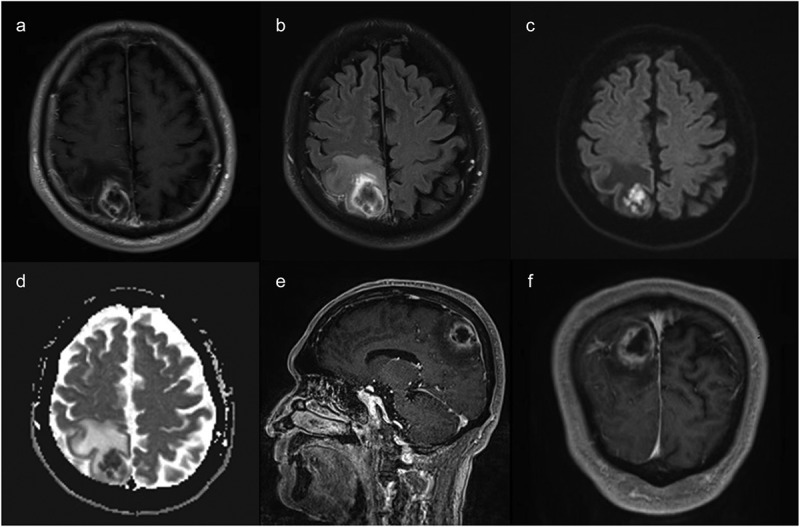
MRI of the brain after admission showed a thin rim-enhancing lesion with perilesional edema in the right parietal lobe (A.B.E.F). The lesion had internal restricted diffusion on DWI and ADC (C.D).

On 25 December 2023, Magnetic resonance imaging (MRI) of the brain showed a thin rim-enhancing lesion (28 mm × 25 mm × 30 mm) with perilesional edema in the right parietal lobe. The lesion had internal restricted diffusion on diffusion-weighted imaging (DWI) and apparent diffusion coefficient (ADC) (Figure 1). Consider the possibility of an acute brain abscess in the right parietal lobe.

Upon admission, the patient was treated with intravenous meropenem (2 g every 8 hours) and linezolid (300 mg every 12 hours) to address the infection. Nine days later, a head CT revealed progression of cerebral edema and a midline shift. Consequently, intravenous dexamethasone (10 mg daily) was introduced to manage the worsening angioedema.

Seventeen days after admission, the patient’s condition stabilized, and he underwent external drainage of a right parietal lobe intracranial abscess via puncture and tube placement under computed tomography guidance. The procedure yielded 18 mL of grayish-white, viscous pus. Pus cultures and mNGS were conducted to identify pathogens and guide appropriate clinical management. The pathogen resulting from culture was *Streptococcus constellatus*. The mNGS results revealed the following relative abundances: *Porphyromonas gingivalis* (7.88%), *S. constellatus* (6.8%), *Filifactor alocis* (0.69%), *Tannerella forsythia* (0.43%) and *Fusobacterium nucleatum* (0.09%). The causative organisms were ultimately identified as *P. gingivalis* and *S. constellatus*. Based on the above results, the final diagnosis was a polymicrobial infectious brain abscess with P. gingivalis and S. constellatus as the primary pathogenic bacteria.

**Figure 2.**
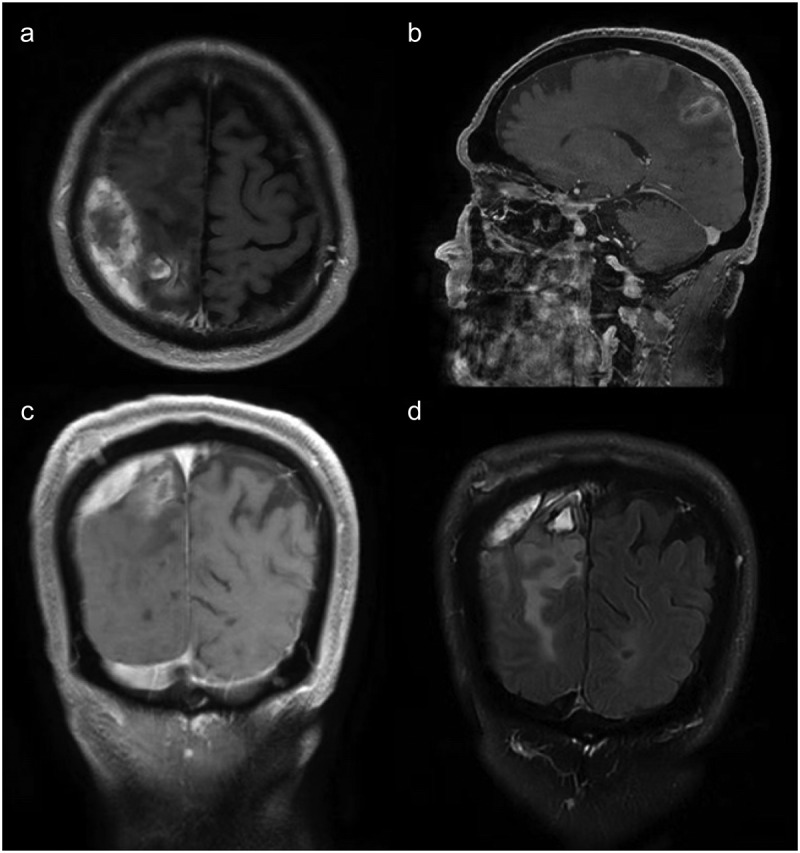
MRI of the brain before discharge (A–D) Showed that the pus cavity in the right parietal lobe had notably narrowed, and peripheral cerebral edema had slightly reduced compared to previous imaging.

The patient continued anti-infective treatment with meropenem (2 g every 8 hours) and linezolid (300 mg every 12 hours) for 2 weeks. Follow-up MRI of the brain revealed significant improvement: the pus cavity in the right parietal lobe had notably narrowed, and peripheral cerebral edema had slightly reduced compared to previous imaging (Figure 2). The patient was alert and discharged in stable condition, with no adverse outcomes.

**Figure 3.**
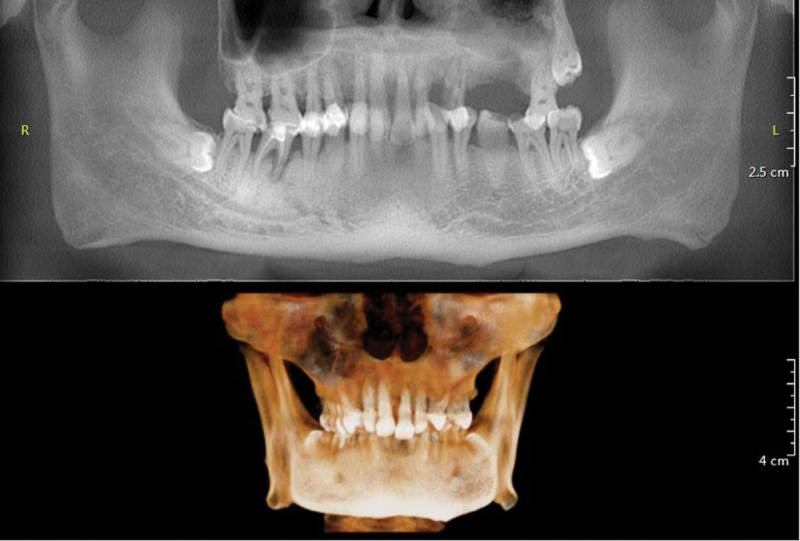
CBCT revealed that the full mouth alveolar bone was absorbed to varying degrees into the middle 1/3 of the root.

**Figure 4.**
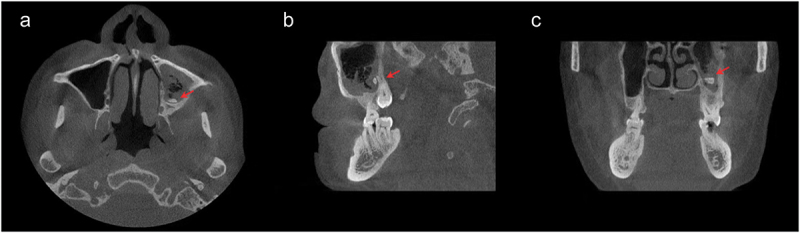
CBCT (A–C) Revealed that a high-density image of a suspected broken tooth in the left maxillary sinus.

On 5 April 2024, two months after discharge, the patient visited the department of stomatology for further examination and treatment. Cone-beam computed tomography (CBCT) of the oral cavity revealed that the full mouth alveolar bone was absorbed to varying degrees into the middle 1/3 of the root. And the mesial root of No. 46 tooth had vertical root fracture with widening of the periradicular lesion surrounding the root (Figure 3). There was thickening of the mucosa in the maxillary sinuses bilaterally with fluid accumulation in the sinus cavities. Meanwhile, the left maxillary sinus was accompanied by a lower wall defect, and a 6 mm *4 mm high-density image of a suspected tooth stump was seen in the sinus (Figure 4). Based on intraoral examination and CBCT imaging, the patient was diagnosed with chronic periodontitis (stage IV, class B), No. 46 tooth vertical root fracture and left maxillary sinus foreign body.

## Discussion

Periodontitis is one of the most common inflammatory diseases globally [6], and the link between periodontal infections and systemic infections is a growing concern. The role of periodontal pathogens in systemic inflammation and organ dysfunction has recently been highlighted in several systemic diseases, including cardiovascular disease [7], hypertension [8], diabetes mellitus [9], rheumatoid arthritis [7], and Alzheimer’s disease [10].

Jeong Rae Yoo et al. proposed three criteria for establishing the diagnosis of odontogenic brain abscess: (a) no alternative source of bacteremia; (b) microbiological finding reveal pathogens typically found in oral microflora; (c) clinical or radiographic signs of active dental or periodontal disease [11–13]. In this case, the patient presented with severe periodontitis and dental stumps within the maxillary sinus. mNGS results of the drainage fluid from the brain abscess revealed a high abundance of *P. gingivalis* and *S. constellatus*, aligning with the diagnostic criteria for an odontogenic brain abscess.

*P. gingivalis* is a Gram-negative, anaerobic bacillus primarily found in the oral cavity. It is recognized as a major contributor to the pathogenesis of periodontitis and is closely associated with pulp necrosis, root tip inflammation, and the formation of gingival sinus tracts [14,15]. Case reports have documented infections caused by *P. gingivalis* in various parts of the body, including otitis media, appendicitis, gas gangrene, thoracic abscesses, and lung abscesses [16]. Rare cases of brain abscesses caused by *P. gingivalis* [17].

*S. constellatus* is a Gram-positive streptococcus of the Streptococcus Agalactiae Group (SAG), which is found commensally in the oral cavity, upper respiratory tract, and gastrointestinal tract. In the reported cases of *S. constellatus* brain abscesses, the routes of infection transmission have included direct seeding of dental infections and haematogenous spread of infective endocarditis or paravertebral abscesses [18–21].

Additionally, several periodontal pathogens were detected in relatively low abundance by mNGS, including *F. alocis*, *T. forsythia* and *F. nucleatum*. It has been suggested that these bacteria may act synergistically with *P. gingivalis* and *S. constellatus* [22–26]. They could potentially serve as biomarkers for identifying the ‘oral-brain axis’.

The ‘oral-brain axis’ remains speculative. However, In cross-sectional studies, many authors have evoked a correlation between the presence of an inflammatory process whose origin could be periodontal and the presence of Alzheimer’s disease (AD) [27,28]. Che et al characterize both the microbial community of subgingival plaque and the metabolomic profiles of gingival crevicular fluid (GCF) in patients with AD and amnestic mild cognitive impairment (aMCI), which revealed that periodontal microbial dysbiosis and metabolic disorders may be involved in the etiology and progression of AD [29]. Frederik et al. found through a population-based cohort study that there is an association between CA with oral microorganisms and the presence of oral pathological conditions, where no other infectious foci in the body could be found [30]. In addition, many studies also have provided evidence for the hypothesis about the ‘oral-brain axis’ [31].

The characteristic triad of headache, fever, and focalization only occurs in 20–30% of cases [32]. Most patients present with only non-specific symptoms, which often leads to delays in diagnosis and treatment [33,34]. In addition, individual scholars have reported unusual manifestations of odontogenic brain abscess, such as the ‘stroke-like syndrome’ reported by Pruettichai et al. [35]. When encountering unexplained infections, consider early diagnosis with mNGS, which is both quicker and more accurate than traditional bacterial culture techniques, enabling more timely and targeted antibiotic therapy.

The etiology of odontogenic brain abscesses remains unclear. Odontogenic infections can theoretically enter the cranial vault via one of four routes: (a) systemic hematogenous bacteremia; (b) direct venous drainage via the two main venous networks leading to the cavernous sinus, the facial and the pterygoid vein systems; (c) inoculation via contiguous extension or by introduction of foreign objects; and (d) lymphatic drainage [5]. The patient’s CBCT indicated that the inflammation from the periodontal infection might have resulted from erosion of the bony plate at the base of the maxillary sinus. Additionally, broken tooth roots retained in the maxillary sinus could have contributed to a septic infection. This infection likely spread through the sinuses, causing erosion and destruction of the cranial bones, ultimately ascending to the brain and leading to the development of a brain abscess [5,36,37]. However, since the brain abscess in this patient was located in the right parietal lobe, not on the side where the tooth stump entered the maxillary sinus, and considering his poor periodontal condition throughout the mouth, the etiology could also be hematogenous [38].

Odontogenic brain abscesses are infrequent, and currently, there are no specific dental strategies established for the treatment of brain abscesses. It is advisable for dentists to perform a proper preoperative assessment as well as prophylactic antibiotics and gentle handling during initial periodontal treatment of immunocompromised patients, in order to prevent transient bacteraemia that could cause or exacerbate brain abscesses. Dentists should be vigilant about the link between a patient’s oral disease and systemic conditions. They should inform the patient of this connection and advise them on the necessity of comprehensive treatment, rigorous oral hygiene practices, and consistent follow-up appointments.

This study has inherent limitations. It relies on single-patient data and therefore has restricted generalizability. Additionally, there is a lack of follow-up imaging data. It would be of great help to closely follow up with the patient as well as increase the number of collected cases in the future.

## Conclusion

This case of an odontogenic brain abscess caused by *P. gingivalis* and *S. constellatus* in a 70-year-old man highlights the critical need to examine the connection between oral flora and systemic health. It also underscores the value of biological sequencing analyses for unexplained infections. Routine and advanced microbiological diagnostics are essential for accurately identifying and effectively managing odontogenic brain abscesses.
